# Depression and Its Determinants Among Patients With Sickle Cell Disease: A Cross‐Sectional Study

**DOI:** 10.1002/puh2.70010

**Published:** 2024-11-25

**Authors:** Bhuwan Dahit, Madhusudan Subedi, Ajay Kumar Rajbhandari, Amit Arjyal, Data Ram Adhikari, Padam Kanta Dahal

**Affiliations:** ^1^ Patan Academy of Health Sciences Lalitpur Nepal; ^2^ Ministry of Health and Population Kathmandu Nepal; ^3^ School of Health, Medical and Applied Sciences Central Queensland University, Sydney Campus Sydney NSW Australia

**Keywords:** depression, low‐resource setting, sickle cell disease, socioeconomic status

## Abstract

**Background:**

Depression among patients with sickle cell disease (SCD) is crucial as it is often underdiagnosed and undertreated. Therefore, this study aimed to determine the prevalence of depression and to identify its determinants.

**Methods:**

This cross‐sectional study was conducted in the Bardiya district of Nepal, based on registered cases of SCD. Depression was measured using the validated Nepalese version of the Beck Depression Inventory (BDI). A random sample of 358 participants with SCD aged >13 years was included in the study. Descriptive and inferential statistics were applied to sociodemographic, clinical and psychological variables. Logistic regression analysis was performed for bivariate analysis. Variables with a *p* value of less than 0.05 were subjected to final multivariate logistic regression.

**Results:**

The prevalence of depression was 36.31% among patients with SCD, age (*p* = 0.0178), education (*p* = 0.0178) and sociodemographic status (*p* = 0.0328) were strongly associated. Factors such as patients aged 40–49 years (adjusted odds ratio [AOR] = 3.05), complications due to SCD (AOR = 1.86) and genetic counselling group (AOR = 4.72) had higher chances of experiencing depression compared to their relative counterparts. However, patients with lower middle‐class economic status, who experience pain crisis, with moderate self‐esteem and who experience discrimination were 52%, 83%, 58% and 58% less likely to have depression compared to their respective counterparts, respectively.

**Conclusions:**

Depression was prevalent in patients with SCD, and multiple sociodemographic, clinical and psychological factors were strongly associated. This warrants the urgent need for early diagnosis and treatment of depression among participants with SCD.

## Graphical abstract

The prevalence of depression was 36.31% among patients with SCD where sociodemographic, clinical and psychological factors were strongly associated.

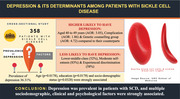

## 1 Introduction

Depression is a major global problem that ranks as the single largest contributor (7.5%) to global disability [1]. The total number of people living with depression worldwide is 322 million, of whom 27% belong to the Southeast Asia region [1]. Similarly, the worldwide prevalence of depression among patients with sickle cell disease (SCD) has been found to be 18%–44% [2, 3].

A study in the United States of America (USA) demonstrated that the prevalence of major depression in SCD patients was 22%, which was significantly associated with a lack of social support, frequent vaso‐occlusive pain crisis, emergency room visits, hospitalizations, disease severity and quality of life in adults [4, 5, 6, 7]. A study in Nigeria estimated that 49.8% of cases were significantly associated with age, marital status, educational level and incurable medical conditions [8]. In the eastern province of Saudi Arabia, the prevalence of depression among SCD patients was 48.2%, which was significantly associated with the lower education level of patients, lower income level, high frequency of vaso‐occlusive crises and frequent visits to haematology clinics [9].

Evidence from previous studies has shown a significant burden of SCD among the Tharu communities in Nepal, with a high possibility of associated mental health issues, including depression. A study by Pande et al. in 2019 showed that the prevalence of SCD among the Tharu communities was 48.08% [10]. Another study by Pandey and Shrestha in 2022 indicated that around 15% of the Tharu patients who had visited the outpatient department had SCD [11]. Further, in these communities, mental health issues, including depression, are most common among SCD patients [12]. Limited resources, constrained health budgets and a lack of psychiatric services are the major contributors to the increasing prevalence of these disorders [13, 14]. The chronic nature of SCD and continuous adherence to treatment regimens diminish the productive livelihood of patients, which also provokes poor mental status [15]. Therefore, it is necessary to determine the prevalence of depression in SCD [16]. To our best knowledge, no study has been published on the prevalence of depression among patients with SCD in Nepal till date. In addition, depression and other mental problems may exist with chronic diseases, and the coexistence of depression among patients with SCD in resource‐poor settings, like Nepal, has not been well elucidated. Thus, this study aimed to determine the prevalence of depression and its determinants among patients with SCD in the Bardiya district of Nepal.

## 2 Methods

This quantitative, community‐based cross‐sectional study was conducted among SCD patients in the Bardiya district of Nepal, following the Strengthening of observational studies in epidemiology (STROBE) guidelines (Supporting Information). Data were collected over a period of 2 months (September 2020–October 2020).

### 2.1 Selection Criteria

Patients aged 13–60 years who were diagnosed with SCD confirmed through a haemoglobin electrophoresis test were included [17]. These patients were registered for subsidies in Barbardiya and Rajapur municipalities of Bardiya district Nepal [18]. The reason for starting at 13 years of age is to focus on adolescents and adults, as they are more likely to experience the symptoms of depression, have emotional maturity and can provide accurate reports. Further, a previous study in Nepal indicated that the mean age of SCD patients was 24.5 ± 12 years [10]. Patients were selected randomly, and those who provided informed consent (i.e., patients aged above 18) and assent (i.e., patients aged below 18) were included in the study. Similarly, patients with SCD who had already been diagnosed with depression and were undergoing treatment were included.

Patients aged <13 years with SCD and those who refused to participate in the study were excluded. Those who could not be contacted during the data collection period were excluded from the study. Informed consent was obtained from all patients, including 51 assent forms.

### 2.2 Sample Size Determination

Because there was no published study for the prevalence of depression among SCD patients in the context of Nepal, a pilot study was done to find out the prevalence for final sample size calculation for this study. The total sample size was calculated assuming a 50% prevalence. Subsequently, 10% of the total sample size, approximately 38.4 (∼40) patients, was used for a pilot study conducted in a similar setting of Tikapur and its interconnected municipalities of the Kailali district of Nepal. Depression among SCD was found at 32.5% with an alpha reliability of 0.7945 using the Nepalese version Beck Depression Inventory (BDI) scale. The single‐proportion sample size formula was used to calculate the required minimum sample size for the study by taking 32.5% prevalence, a 5% margin of error and a 10% non‐response rate to make the final sample size. The final sample size considered for this study was 371 participants by the formula of *n* = (*z* ^2^ *pq*/*d* ^2^) × (1 + non‐response) [19]. Here, *n* = total sample size, *p* = 0.325, *q* = 0.675, *z* = 1.96, *d* (allowable error) = 0.05 at 95% CI, non‐response rate = 10% = 0.1.

### 2.3 Sampling Method

The Barbardiya and Rajapur municipalities of Bardiya district were considered purposively in this study because the majority of these sites are home to Indigenous Tharu communities. All patients meeting the inclusion criteria were randomly chosen for data collection to ensure a representative sample and reduce selection bias. This involved listing all eligible patients and then randomly selecting patients from this list, ensuring that each patient had an equal chance of being included in the study.

### 2.4 Study Tools and Outcome Variables

We conducted one‐on‐one phone interviews using structured questionnaires for data collection. The survey questionnaires were standard tools that were already pre‐validated in Nepal, with an extensive literature review incorporating expert opinions, including consultant physicians and psychiatrists. The validated BDI‐Nepali questionnaire was used to determine the prevalence of depression among patients with SCD [20]. The modified Kuppuswamy's socioeconomic status scale was used to assess socioeconomic status [21]. Questionnaires prepared from an extensive literature review were used to identify clinical, psychological and social factors associated with depression, which were validated through a pilot study conducted as a part of this study in similar community settings in the Kailali district of Nepal. Local validation of all compiled tools was performed among 40 individuals with SCD in the Kailali district, and necessary modifications were made according to the requirements of the research objectives. In addition, the Nepali version of the Rosenberg 10‐item scale adopted from the Transcultural Psychological Organization (TPO) was used to measure self‐esteem as a factor of depression [22].

Depression among patients with SCD is a primary outcome variable. Further, its associated factors, including background and mediating variables, are secondary outcomes of this study. Background variables consisted of sociodemographic and economic status, which were measured on the basis of the educational and occupational status of the household head and income according to the modified Kuppuswamy scale. The mediating variables were the clinical and psychological factors. Clinical factors include painful crises, multiple blood transfusions, emergency visits, hospitalization and complications in SCD. Psychological factors included self‐esteem, genetic counselling and discrimination.

### 2.5 Statistical Analysis

Data were analysed using Epi Info 7 (for data entry and data cleaning) and Easy R (EZR) version 3.5.2 (for data analysis). Descriptive and inferential statistics were used to determine the prevalence of depression and its determinants in patients with SCD. Descriptive statistics, such as percentage, frequency and mean, were computed to determine the prevalence of depression according to sociodemographic, clinical and psychological variables. Variables with a *p* value of <0.05 at the unadjusted model were taken to the adjusted model. Logistic regression was used to test the interactions among the various associated variables. Statistical significance in the final model was set at *p* < 0.05.

## 3 Results

### 3.1 Background Characteristics of the Study Participants

Table 1 shows the distribution of the patients according to their demographic and psychological characteristics. The mean age of the patients was 28.92 (±0.53) years, and the majority (37.99%) were aged between 20 and 29 years. Of the total patients, 15.92% were illiterate and approximately 32% were students. Six major subgroups of Tharu were identified, among which the majority (45.53%) were Deshuri Tharu. One out of three patients, 36.31% (95% CI; 31.32–41.53), had daily depressive mood disorder for more than 2 weeks. Of the total patients, 13.97%, 69.27% and 16.76% had low, moderate and high self‐esteem, respectively. Nearly half of the patients (*n* = 241) partially understood, which was explained in front of patients at genetic counselling sessions, and more than half (60.61%) felt discriminated against because of their condition.

**TABLE 1:** Sociodemographic characteristics of the study participants.

Variables	Frequencies (*n* = 358)	Percentage (%)
Median age: 28 years		
Age group		
13–19	63	17.60
20–29	136	37.99
30–39	108	10.17
40–49	37	10.34
50–59	14	3.91
Sex		
Male	189	52.79
Female	169	47.21
Marital status		
Never married	132	36.87
Married	210	58.66
Separated/Divorced/Widowed	16	4.47
Education		
No education	57	15.92
Primary education	80	22.35
Secondary	179	50
Higher secondary and above	42	11.73
Current occupation		
Government employee	4	1.12
Non‐government employee	14	3.91
Self‐employed	79	22.07
Non‐paid	21	5.87
Student	114	31.84
Construction	100	27.93
Unemployed (able to work)	21	5.87
Unemployed (unable to work)	5	1.40
Subgroup of Tharu		
Rana/Lampucchwa Tharu	9	2.52
Kathariya Tharu	44	12.29
Sonha Tharu	12	3.35
Dangaura Tharu	130	36.31
Deshuri Tharu	163	45.53
Socioeconomic status		
Upper middle class	17	4.75
Lower middle class	58	16.20
Upper lower class	234	65.36
Lower class	49	13.69
Prevalence of depression		
No depression	228	63.69
Depression	130	36.31
Psychological characteristics		
Self‐esteem		
Low	50	13.97
Moderate	248	69.27
High	60	16.76
Genetic counselling		
Yes	241	67.32
No	117	32.68
Understood sessions		
Yes	73	30.29
Partially yes	107	44.40
No	61	25.31
Discrimination		
Yes	217	60.61
No	141	39.39

### 3.2 Depression and Sociodemographic Variables

The relationships between depression and sociodemographic characteristics are summarized in Table 2. Age (i.e., *p* = 0.0178), education (i.e., *p* = 0.0178) and socioeconomic status (i.e., *p* = 0.0328) were significantly associated with depressive symptoms. In the adjusted model, participants aged 40–49 years had 3.05 (adjusted model, adjusted odds ratio [AOR]: 3.05; 95% CI; 1.05–8.89, *p* = 0.0411) times more likely to experience symptoms of depression compared to those aged 13–19 years old. Further, those in the upper lower economic class were 52% (adjusted model, AOR: 0.48; 95% CI; 0.66–6.03, *p* = 0.0132) less likely to have depression compared to the upper middle class.

**TABLE 2:** Association between depression and sociodemographic characteristics.

Sociodemographic characteristics	Depression		Unadjusted analysis		Adjusted analysis^*^	
	Yes (%)	No (%)	OR (95% CI)	*p* value	AOR (95% CI)	*p* value
Age group			***p* value = 0.0178**			
13–19 [Ref]	16 (25.40)	47 (74.60)	1		1	—
20–29	58 (42.65)	78 (57.35)	2.18 (1.13–4.23)	0.0206	1.46 (0.64–3.32)	0.3620
30–39	31 (28.70)	77 (71.30)	1.18 (0.58–2.39)	0.6410	0.65 (0.27–1.58)	0.3480
40–49	19 (51.35)	18 (48.65)	3.10 (1.31–7.32)	0.0098	3.05 (1.05–8.89)	0.0411
50–59	6 (42.86)	8 (57.14)	2.20 (0.66–7.32)	0.1970	0.78 (0.17–3.55)	0.7500
Sex			***p* value = 0.763**			
Male [Ref]	70 (37.04)	119 (62.96)	1	—	—	—
Female	60 (35.50)	109 (64.50)	0.93 (0.60–1.44)	—	—	—
Marital status			***p* value = 0.1890**		—	—
Never married [Ref]	42 (31.82)	90 (68.18)	1	—	—	—
Married	84 (40)	126 (60)	1.43 (0.90–2.26)	—	—	—
Separated/Divorced/Widowed	4 (25.00)	12 (75.00)	0.71 (0.22–2.35)	—	—	—
Educational status			***p* value = 0.0178**			
No education [Ref]	28 (49.12)	29 (50.88)	1		1	
Primary education	25 (31.25)	55 (68.75)	0.47 (0.23–0.95)	0.0355	0.81 (0.30–2.13)	0.6710
Secondary	56 (31.28)	123 (68.72)	0.47 (0.25–0.86)	0.0154	0.72 (0.31–1.63)	0.4350
Higher secondary and above	21 (50)	21 (50)	1.04 (0.46–2.30)	0.9310	2.0 (0.66–6.03)	0.2210
Current occupation			***p* value = 0.7473**		—	—
Government employee [Ref]	1 (25)	3 (75)	1	—	—	—
Non‐government employee	6 (42.86)	8 (57.14)	2.25 (0.18–27.4)	0.525	—	—
Self‐employed	32 (40.41)	47 (59.49)	2.04 (0.20–20.5)	0.544	—	—
Voluntary	6 (28.57)	15 (71.43)	1.20 (0.10–14.0)	0.884	—	—
Student	36 (31.58)	78 (68.42)	1.38 (0.13–13.8)	0.781	—	—
Housemaker	41 (100)	59 (59)	2.08 (0.20–20.8)	0.531	—	—
Unemployed (able to work)	7 (33.33)	14 (66.67)	1.50 (0.13–17.2)	0.744	—	—
Unemployed (unable to work)	1 (20)	4 (80)	0.75 (0.03–17.5)	0.858	—	—
Subgroup of Tharu			***p* value = 0.8777**		—	—
Rana Tharu/Lampucchwa Tharu [Ref]	3 (33.33)	6 (66.67)	1		—	—
Kathariya Tharu	15 (34.09)	29 (65.91)	1.03 (0.23–4.73)	0.965	—	—
Sonha Tharu	4 (33.33)	8 (66.67)	1.00 (0.16–6.26)	1.000	—	—
Dangaura Tharu	52 (40)	78 (60)	1.33 (0.32–5.57)	0.693	—	—
Deshuri Tharu	56 (34.36)	107 (65.64)	1.05 (0.25–4.34)	0.950	—	—
Socioeconomic status			***p* value = 0.0328**		—	—
Upper middle class [Ref]	9 (52.94)	8 (47.06)	1		1	
Lower middle class	29 (50)	29 (50)	0.88 (0.30–2.62)	0.8310	0.48 (0.12–1.88)	0.2930
Upper lower class	74 (31.62)	160 (68.38)	0.41 (0.15–1.11)	0.0789	0.48 (0.12–1.88)	0.0132
Lower class	18 (36.73)	31 (63.27)	0.51 (0.16–1.57)	0.2450	0.25 (0.06–1.06)	0.0595

Abbreviations: AOR, adjusted odds ratio; OR, odds ratio.

* *p* value of less than 0.05 for different predictors was used in the final model, and the value obtained from the final model.

### 3.3 Depression and Clinical Variables

The associations between depression and the clinical factors are shown in Table 3. In adjusted model, participants with SCD complications in SCD had 1.86 (adjusted model, AOR: 1.86; 95% CI; 1.06–3.26, *p* = 0.0299) times more likely to develop depression than those with no complications attributable to SCD. Further, patients who experience pain crises are 83% (adjusted model, AOR: 0.17: 95% CI; 0.04–0.63, *p* = 0.0084) less likely to have depression compared with the absence of a pain crisis.

**TABLE 3:** Association between depression and clinical factors.

Clinical factors	Depression		Unadjusted analysis		Adjusted analysis^*^	
	Yes (%)	No (No. %)	OR (95% CI)	*p* value	AOR (95% CI)	*p* value
Emergency visit or hospital admission			***p* value = 0.0680**		—	—
No [Ref]	36 (30)	84 (70)	1		1	
Yes	94 (39.50)	144 (60.50)	1.60 (0.96–2.66)	0.0680	1.13 (0.59–2.15)	0.7140
Blood transfusion over the past 12 months			***p* value = 0.0680**			
No [Ref]	94 (33.81)	184 (66.19)	1		1	—
Yes	36 (45)	44 (55)	1.60 (0.96–2.66)	**0**.0680	1.01 (0.49–2.07)	0.9680
Complication(s) attributable to sickle cell disease			***p* value = 0.0020**		—	—
No [Ref]	48 (28.07)	123 (71.93)	1		1	
Yes	82 (43.85)	105 (56.15)	2.00 (1.29–311)	0.0020	1.86 (1.06–3.26)	0.0299
Pain crisis			***p* value = 0.0332**		—	—
No [Ref]	10 (62.50)	6 (37.50)	1		1	—
Yes	120 (35.09)	222 (64.91)	0.32 (0.11–0.91)	0.0332	0.17 (0.04–0.63)	0.0084

Abbreviations: AOR, adjusted odds ratio; OR, odds ratio.

* *p* value of less than 0.05 for different predictors was taken to the final model, and the value obtained from the final model.

### 3.4 Depression and Psychological Variables

The relationship between depression and the psychological variables is shown in Table 4. In the adjusted model, patients with moderate self‐esteem had 58% (adjusted model, AOR: 0.42, 95% CI; 0.19–0.94, *p* = 0.0368) less likely to have depression compared to those with low self‐esteem. The genetic counselling group had 4.72 (adjusted model, AOR: 4.72, 95% CI; 2.37–9.39, *p* = 0.0001) times more likely to experience depression than those who had not been counselled. Interestingly, depression among those who had experienced discrimination due to their health condition was 58% (adjusted model, AOR: 0.42, 95% CI; 0.23–0.76, *p* = 0.0039) less likely compared to those who did not.

**TABLE 4:** Association between depression and psychological factors.

Psychological factors	Depression		Unadjusted analysis		Adjusted analysis^*^	
	Yes (No. %)	No (No. %)	OR (95% CI)	*p* value	AOR (95% CI)	*p* value
Self‐esteem type			***p* value = 0.0434**		—	—
Low [Ref]	26 (52)	24 (48)	1	—	1	—
Moderate	82 (33.06)	166 (66.94)	0.45 (0.24–0.84)	0.0123	0.42 (0.19–0.94)	0.0368
High	22 (36.67)	38 (63.33)	0.53 (0.24–1.15)	0.1080	0.60 (0.22–1.62)	0.3180
Genetic counselling			***p* value = 1.19e–06**		—	—
No [Ref]	21 (17.95)	96 (82.05)	1	—	1	—
Yes	110 (45.23)	132 (54.77)	3.77 (2.21–6.45)	1.19e–06	4.72 (2.37–9.39)	0.0001
Discrimination			***p* value = 0.0082**		—	—
No [Ref]	63 (44.68)	78 (55.32)	1	—	1	—
Yes	67 (30.88)	150 (69.12)	0.55 (0.35–0.85)	0.0082	0.42 (0.23–0.76)	0.0039

Abbreviations: AOR, adjusted odds ratio; OR, odds ratio.

* *p* value of less than 0.05 for different predictors was taken to the final model and the value obtained from the final model.

## 4 Discussion

This study identified the prevalence of depression among participants with SCD and explored its associated factors, including sociodemographic, clinical and psychological factors. The current study indicated that the prevalence of depression is 36.31%, which is significantly higher among the participants with SCD. Furthermore, this study found that depression was significantly associated with sociodemographic (i.e., age and socioeconomic status), clinical (i.e., participants who had complications and pain) and psychological (i.e., participants with moderate self‐esteem, experienced discrimination and genetic counselling group) factors among participants with SCD.

The present study found that the prevalence of depression among patients with SCD was 10% higher than in the previous systematic review study by Doglioni et al. in 2021 [23]. This systematic review identified that the average prevalence of depression among adult patients with SCD was 24.01% (ranging from 0% to 85.90%). In Middle Eastern countries, the prevalence of depression among patients with SCD was explored through some cross‐sectional studies, which were proven to be higher (i.e., 58.5% in Bahrain [24] and 85.9% in Saudi Arabia [25]) than in the current study (36.31%). Furthermore, the prevalence of depression in SCD was 68.2% in Uganda [26] and 35.2% in the USA [27]. These differences may be due to a low level of education, comorbid conditions and low social support [14]. This is because the lower level of education may limit the understanding and awareness of the disease and reduce the likelihood of seeking support and receiving treatment [28]. Further, the presence of comorbidity with SCD may exacerbate mental health problems, including depression, resulting higher prevalence of depression [29]. Patients with a lack of social support may feel isolated leading to a higher depression rate in the different regions [30]. Additionally, variations in healthcare resources in different regions and settings may influence the depression rates [31].

Similar to previous studies [7, 32], our current study identified that sociodemographic factors, such as age, education and socioeconomic status, were significantly associated with depressive symptoms. Further, in the adjusted analysis, the age group of 40–49 years had a three times higher likelihood of having depression and the upper lower class socioeconomic group was over 50% less likely to have depression compared to the upper middle‐class group. This might be due to lower awareness, limited financial resources, lack of social support and higher stigma towards mental health issues, preventing individuals from seeking help [33, 34]. In contrast, a study conducted in Uganda in 2018 showed that high depression was associated with low socioeconomic status [26]. These differences might be due to differences in education levels, work preferences and economic differences across different regions [14, 33].

Depression was less common among pain crises attributable to SCD (AOR = 0.17). A similar study conducted in Saudi Arabia showed that those who had experienced a pain crisis due to SCD had fewer chances of depression (AOR = 0.17) [9]. In contrast, an analytical epidemiologic prospective study in the United States in 2017 showed poor compliance between depression and pain crisis [27, 34]. These differences may be due to methodological issues such as regular recording and reporting of pain crises in these studies [23].

Our study found that self‐esteem, genetic counselling and discrimination are associated with depression among SCD patients. Patients with moderate self‐esteem had significantly lower chances of having depression compared to those with low self‐esteem group. This finding is similar to the findings from a systematic review by Kilonzi et al. in 2022, which shows that patients with SCD and their families reduce the self‐confidence in their life [35]. Furthermore, this study identified that depression was five times higher among those who attended genetic counselling (AOR = 4.72), which contradicts a qualitative study performed in Ghana in 2020 that suggested that genetic counselling helps decrease the burden of diseases [36]. This difference in findings might be due to a lack of understanding of the genetic counselling sessions. In addition, our study showed that depression was less common among those who had experienced discrimination due to SCD (AOR = 0.42). In contrast, a qualitative study in the United Kingdom in 2016 revealed that people living with SCD experience discrimination and depression as an illness [37]. These divergent results suggest that qualitative studies are needed to illustrate the impact of discrimination among SCD patients in community settings.

Our study has some strengths and limitations. Initially, this study provides the prevalence, and factors associated with depression among SCD patients, a topic that is new to Nepalese indigenous community settings. It offers insights into a population that is often underrepresented in research and those with positive depression were advised to seek further interventions and referred to mental health professionals for appropriate treatment and counselling. Then, our findings provide a holistic understanding of the mental health challenges faced by the Tharu communities. Further, by focusing on these communities, our results can inform tailored interventions and support systems to reduce the prevalence of SCD in the targeted areas. Additionally, the study employs validated and reliable tools for data collection, enhancing the credibility and accuracy of the findings. However, our study has some limitations. First, due to travel constraints posed by the COVID‐19 pandemic, it was decided to sample two large municipalities where restrictions had been lifted, rather than proportionally sampling all municipalities in the Bardiya district. This may have overlooked some of the differences in the prevalence of and factors associated with depression in the entire district. Second, there was a higher chance of recall bias, leading to differences in accuracy or completeness by the study participants regarding past events or experiences. Third, the association between depression levels and various other factors such as financial stress, chronic pain, individual disease comorbidity, family conflict, divorce, loss of job and physical impact was not included in the analysis. Finally, biopsychological factors such as genetic predisposition, anaemia, medication effects, inflammation, coping mechanisms, previous mental health history and stigma for depression were not considered determinants in this study because of time constraints.

## 5 Conclusion

In conclusion, the prevalence of depression among patients with SCD was higher in a community setting, which is alarming in Nepal. Furthermore, clinical and psychological factors were associated with depression among SCD patients and are essential for the early detection and timely management of depression.

The arrangement of comprehensive and prompt clinical management of SCD complications as part of an essential healthcare package might be effective for disease management in a community setting with limited healthcare resources. Strengthening local healthcare workers and facilities with proper training in psychological care can also improve mental health issues, including depression among SCD patients. In addition, further research in a similar setting on a large scale is needed to generate evidence and design appropriate interventions for SCD management.

## Author Contributions

**Bhuwan Dahit**: conceptualization, methodology; software, writing–original draft, visualization, formal analysis, writing–review and editing. **Madhusudan Subedi**: conceptualization, supervision, writing–review and editing, visualization, methodology. **Ajay Kumar Rajbhandari**: conceptualization, software, writing–review and editing, supervision, data curation. **Amit Arjyal**: methodology, writing–review and editing, writing–original draft, formal analysis, supervision. **Data Ram Adhikari**: methodology, investigation, supervision, formal analysis, writing–original draft, data curation, writing–review and editing. **Padam Kanta Dahal**: writing–original draft, methodology, writing–review and editing, software, conceptualization, visualization, formal analysis, validation, supervision.

## Consent

Informed consent was obtained from all patients, including 51 assent forms

## Ethics Statement

This study was approved by the Institutional Review Committee of the Patan Academy of Health Sciences, Lalitpur, Nepal (Reference: PHP2008251436), and a consent letter was obtained from the Barbardiya and Rajapur Municipality of Nepal. Informed consents were obtained from all participants, including 51 assent forms. This study was conducted in accordance with the guidelines outlined in the Helsinki Declaration. Both informed consent and assent forms (i.e., who were aged under 18) were obtained from the participants. This study was conducted in accordance with the guidelines outlined in the Helsinki Declaration.

## Conflicts of Interest

The authors declare no conflicts of interest.

## Supporting information



Supporting Information

## Data Availability

Data will be available on request from the authors.
